# The Maintenance of Traditions in Marmosets: Individual Habit, Not Social Conformity? A Field Experiment

**DOI:** 10.1371/journal.pone.0004472

**Published:** 2009-02-18

**Authors:** Mario B. Pesendorfer, Tina Gunhold, Nicola Schiel, Antonio Souto, Ludwig Huber, Friederike Range

**Affiliations:** 1 Department of Neurobiology and Cognition Research, University of Vienna, Vienna, Austria; 2 Departamento de Biologia, Área Zoologia, Universidade Federal Rural de Pernambuco, Recife, Pernambuco, Brazil; 3 Departamento de Zoologia, Centro de Ciências Biológicas, Universidade Federal de Pernambuco, Recife, Pernambuco, Brazil; University of Pretoria, South Africa

## Abstract

**Background:**

Social conformity is a cornerstone of human culture because it accelerates and maintains the spread of behaviour within a group. Few empirical studies have investigated the role of social conformity in the maintenance of traditions despite an increasing body of literature on the formation of behavioural patterns in non-human animals. The current report presents a field experiment with free-ranging marmosets (*Callithrix jacchus*) which investigated whether social conformity is necessary for the maintenance of behavioural patterns within groups or whether individual effects such as habit formation would suffice.

**Methods:**

Using a two-action apparatus, we established alternative behavioural patterns in six family groups composed of 36 individuals. These groups experienced only one technique during a training phase and were thereafter tested with two techniques available. The monkeys reliably maintained the trained method over a period of three weeks, despite discovering the alternative technique. Three additional groups were given the same number of sessions, but those 21 individuals could freely choose the method to obtain a reward. In these control groups, an overall bias towards one of the two methods was observed, but animals with a different preference did not adjust towards the group norm. Thirteen of the fifteen animals that discovered both techniques remained with the action with which they were initially successful, independent of the group preference and the type of action (Binomial test: exp. proportion: 0.5, p<0.01).

**Conclusions:**

The results indicate that the maintenance of behavioural patterns within groups 1) could be explained by the first rewarded manipulation and subsequent habit formation and 2) do not require social conformity as a mechanism. After an initial spread of a behaviour throughout a group, this mechanism may lead to a superficial appearance of conformity without the involvement of such a socially and cognitively complex mechanism. This is the first time that such an experiment has been conducted with free-ranging primates.

## Introduction

The emergence of culture through preservation of multiple traditions is considered a hallmark of human evolution as it creates an inheritance system that is largely independent of genetic transmission [1], [2]. Cultural phenomena were thought to be limited to humans. However, over recent years, a number of studies have claimed that they have demonstrated similar phenomena in other vertebrate species. For example, different chimpanzee (*Pan troglodytes*) communities display a suite of traditions, creating a culture that uniquely defines each community [3], [4]. A tradition has been defined as a distinctive behaviour pattern shared by two or more individuals in a social unit that persists over time and that new practitioners may acquire in part through socially aided learning [5]. Behavioural innovations and their spread can have significant effects on the fitness of individuals in a group if they do or do not partake in a new behavioural practice [6], as such practices can contribute to niche construction through behavioural adaptations to environmental changes [7].

There is an ongoing and lively discussion about the definitions and methods used to elucidate different aspects of socially transmitted behaviour in regard to culture and/or traditions [8]–[10]. Using the ethnographical approach of contrasting different populations of the same species, observational studies have identified behaviour patterns which vary between populations that are presumed to be independent of ecological and genetic influences (e.g. chimpanzees: [3], macaques: [11], capuchins: [12], cetaceans: [13], corvids: [14], for review see [15]). A weakness of this approach arises from the fact that the origin of the observed behavioural differences remains obscure and that the processes involved in establishing the behaviour are hard to reconstruct [9], [10]. In these studies social transmission of an initial innovation has been inferred on the basis of the geographical distribution of the trait or by mathematical modelling of the studied behaviour [16], but logistical and ethical problems have hindered proper experimentation in wild populations of animals [17]. Experimentation with free-ranging groups of animals is a crucial step in gaining more insight into traditional behaviour [8]. This was one of our main motivations for conducting this study.

Several other studies have approached the topic experimentally in the laboratory by allowing novel skills to spread throughout captive populations (see [18] for review). Beyond describing the vertical and horizontal diffusion, many studies have focused on the social learning mechanisms involved, and sophisticated laboratory experiments have revealed that no distinctive form of social learning is unique to humans and their most closely related primates as often suggested [19], [20]. Common criticism about the laboratory work includes calls for more ecologically relevant paradigms, concerns about (cognitively) impoverished living conditions and creation of behavioural artefacts due to unnatural social conditions [21].

Despite an impressive number of studies on social learning and potential traditions in non-human animals, one enticing aspect has been largely disregarded. After the innovation and initial spread of a novel behaviour, the behaviour stabilizes and is maintained over time. Despite theoretical considerations of the mechanisms involved in maintaining a behaviour in a group that have been formulated twenty years ago [22], [23], little research investigated their application to non-human animals. Research with humans and theoretical models suggest that social conformity, expressed as an exaggerated preference for behaviour displayed by the majority (50% +1) of a group, plays an important role in homogenising and maintaining group behaviour over time [24].

A recent study by Whiten and colleagues [17] conducted with three groups of captive chimpanzees claims that some animals displayed the “tendency to discount personal experience in favour of adopting perceived community norms” (p. 738). Their claim is based on the fact that most animals that had discovered both possible techniques of a two-action task in the first testing session showed a stronger preference for the action that was predominantly used by the other members of that group when they were tested again two months later. While the study shows that a group of chimpanzees acquires and maintains a tool-using tradition over time, it was not specifically designed to test the mechanism involved. The shift in preference towards the social norm could have arisen from either individual or social factors and the design does not allow us to distinguish between these possibilities. More recent work by the first author of that study even concludes “that young chimpanzees exhibit a tendency to become ‘stuck’ on a technique they initially learn, inhibiting cumulative social learning and possibly constraining the species' capacity for cumulative cultural evolution” [25]. Furthermore, the exaggerated preference for a certain behaviour displayed by the majority of a group simply cannot be assumed based on only two animals that change their overall preference for an action, while the rest of the animals decrease their personal preference by a median of 1.13% (interquartile range: 0.39–9.50%, calculated from data in the supplementary information of [17]) towards the group norm.

Studies by Galef & Whiskin [26]–[27] showed that rats (*Rattus norvegicus*) change from an individually learned preference to a socially mediated preference in food choice paradigms in a one-to-one setting. Yet, this is also not conclusive evidence for social conformity, as none of the rats were tested in a group setting where the majority of animals has one preference and the minority of non-conformists have another. Therefore, as alternative explanations to social conformity have not been tested and cannot be excluded, the mechanisms maintaining a tradition in non-human animals remain unclear.

We chose to address this problem with common marmosets, as they display all requirements that suggest that they have the potential to establish traditions. They are a species that are highly sensitive to social information [28]–[34], that occupy stable home ranges, and have been found to live in family groups of six to 15 individuals in our study site [28]. The specific population of marmosets, that has been subject to multiple other studies [28], [35]–[37], consists of 10 or more groups that are accessible and well habituated to human contact, convincing us that it would provide an optimal system to introduce artificial behavioural patterns in several different groups. No similar work has previously been conducted with wild primates, despite a recent call for more field experiments dealing with social phenomena [8].

Combining field work with an experimental approach, our study set out to specifically test whether initial personal preferences for one of two methods are maintained over time or whether they are adapted to the group norm. As the focus of this study was not the initial phase of transmission and learning of a behavioural tradition, but the maintenance of a learned skill in a group, we chose to simplify the procedure by training all members of the group, rather than just a single model. The cognitive mechanism involved in the acquisition of the task is the same as in a social setting, only the source of the information differs.

In order to test the stability of a learned behaviour in the presence of alternative solutions, we established a group norm by presenting an apparatus that could be opened either by pushing or pulling a door [29] to six free-living family groups. In all cases, one of the two actions was blocked during the training phase (“constrained condition”). Three of the groups were only able to pull the door and three were only able to push the door. After the training phase, the six groups were then tested in two test blocks with both methods available. The first test block of three sessions was administered immediately after training and the second test block was conducted 21 days later. During the test phase, our experimental design allowed us to assess i) whether a learned preference established at the group level would remain stable over time, thus simulating a tradition and ii) whether individuals that were suddenly confronted with two available methods in the test block would show a clear preference for the learned method or whether they would switch towards the newly available method. Thus, this condition investigated whether a ‘tradition’ in a group could be established without invoking higher cognitive processes such as social conformity. In addition, these constrained conditions resembled a scenario in which ecologically different situations may have triggered the formation of varying traditions in geographically separated populations. Three additional family groups received the same amount of sessions as the constrained condition groups did during the training, but with the unconstrained apparatus (“free condition”). This situation aimed at elucidating i) whether a group norm of preferentially using one of the two opening methods would be established freely and ii) whether an individual would develop a habit, independent of the dominant method used by the majority of the group, or iii) if it would reverse its preference and conform to the group norm as time went on. Finally, the free condition served to control for a general preference for one of the two actions in this population of marmosets.

## Results

### Constrained Condition Training

Thirty of the thirty-six animals in the six different groups participated in the training sessions of the constrained condition (pull: n = 17; push: n = 13; Tab. S1). Two animals that initially participated (AIS: pull; MAT: push), disappeared during the course of the experiment and were not seen again. To assess the individual acquisition of the skill, we tested whether the efficiency to open the box increased during the training session. We calculated the number of successful actions divided by all actions at the box for the first six and the last six training sessions and compared them. Twenty-four of the twenty-six animals who successfully participated in both the first and the second half of training improved their efficiency over time (Wilcoxon matched-pair test for average efficiency scores: training sessions 1–6 vs. sessions 7–12, n = 26, Z = 4.26, p<0.0001). In five of the six groups, juveniles or subadults were the first individuals to manipulate the box successfully (Tab. S1).

### Constrained Condition Tests

**Figure 1:**
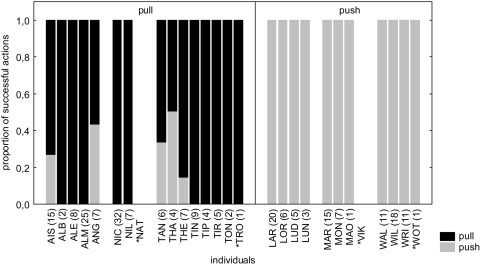
First test block (tests 1–3). Proportion of successful actions push (grey bars) and pull (black bars) in constrained groups. Pull groups are shown on the left side, push groups on the right side. Digits in brackets indicate number of successes. Asterisks mark animals that performed first successful manipulations during test sessions.

**Figure 2:**
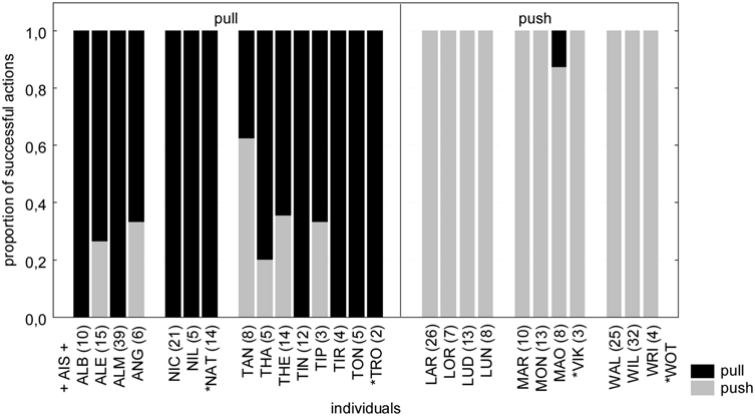
Second test block (tests 4–6). Proportion of successful actions push (grey bars) and pull (black bars) in constrained groups. Pull groups are shown on the left side, push groups on the right side. Digits in brackets indicate number of successes. Asterisks mark animals that performed first successful manipulations during test sessions. “AIS” disappeared before the second test block.

Pulling or pushing traditions were successfully established in all constrained condition groups. In the test sessions, all six groups showed a strong preference (expressed as percentage of one action type in relation to all performed actions) for the trained action and maintained this preference over time (group preference avg. and SD: push: 99.03±0.07%, n = 12; pull: 86.96±0.17%, n = 16, Tabs. 1 and 2, Figs. 1 and 2). Twenty-three of the twenty-nine animals that successfully participated in the second test block showed a preference above 90% for the trained action (Binomial test: n = 29, exp. proportion = 0.5, p = 0.0002). To assess whether the individual preference changed between the test blocks, we compared the preference for the trained action of the first and the second test blocks. The difference between the preferences of the first and second test blocks was not significant in either condition (Push: Wilcoxon matched-pair test: n = 12, T = 5.00, p = 1.0; pull: Wilcoxon matched pair test: n = 14, T = 9.00, p = 0.21).

**Table 1:** Test constrained groups: push.

Individual	All Actions^a^								Successful Actions^b^							
	T1	T2	T3	T4	T5	T6	B1^c^	B2^d^	T1	T2	T3	T4	T5	T6	B1^c^	B2^d^
LAR	1.00	1.00	1.00	1.00	1.00	1.00	**1.00**	**1.00**	1.00	1.00	1.00	1.00	1.00	1.00	**1.00**	**1.00**
LOR	1.00	1.00	1.00	1.00	1.00	1.00	**1.00**	**1.00**	1.00	1.00	1.00	1.00	1.00	1.00	**1.00**	**1.00**
LRZ	n/a	1.00	n/a	1.00	n/a	n/a	**1.00**	**1.00**	n/a	n/a	n/a	n/a	n/a	n/a	**n/a**	**n/a**
LUD	0.90	1.00	1.00	1.00	1.00	1.00	**0.97**	**1.00**	1.00	1.00	1.00	1.00	1.00	1.00	**1.00**	**1.00**
LUN	1.00	1.00	1.00	1.00	1.00	1.00	**1.00**	**1.00**	1.00	n/a	1.00	n/a	1.00	1.00	**1.00**	**1.00**
Avg.^e^							0.99	1.00							1.00	1.00
MAR	0.89	1.00	1.00	0.80	1.00	n/a	**0.96**	**0.90**	1.00	1.00	1.00	1.00	1.00	n/a	**1.00**	**1.00**
MON	1.00	1.00	0.80	1.00	1.00	1.00	**0.93**	**1.00**	1.00	1.00	1.00	1.00	1.00	1.00	**1.00**	**1.00**
MAO	n/a	n/a	1.00	0.80	n/a	0.88	**1.00**	**0.84**	n/a	n/a	1.00	1.00	n/a	0.80	**1.00**	**0.90**
VIC	n/a	n/a	n/a	n/a	1.00	0.57	**n/a**	**0.79**	n/a	n/a	n/a	n/a	1.00	1.00	**n/a**	**1.00**
Avg.^e^							0.97	0.88							1.00	0.98
WAL	1.00	1.00	1.00	1.00	1.00	1.00	**1.00**	**1.00**	1.00	1.00	1.00	1.00	1.00	1.00	**1.00**	**1.00**
WILL	1.00	1.00	1.00	1.00	1.00	1.00	**1.00**	**1.00**	1.00	1.00	1.00	1.00	1.00	1.00	**1.00**	**1.00**
WIR	1.00	1.00	1.00	n/a	1.00	n/a	**1.00**	**1.00**	1.00	n/a	1.00	n/a	1.00	n/a	**1.00**	**1.00**
WOT	n/a	n/a	1.00	n/a	n/a	n/a	**1.00**	**n/a**	n/a	n/a	1.00	n/a	n/a	n/a	**1.00**	**n/a**
Avg.^e^							1.00	1.00							1.00	1.00
**Overall** f							**0.98**	**0.96**							**1.00**	**0.99**

a Proportion of push actions in all actions.

b Proportion of push actions in successful actions.

c Proportion of push actions for block 1: T1–T3.

d Proportion of push actions for block 2: T4–T6.

e Group average for blocks.

f Average across all animals in push condition.

**Table 2:** Test constrained groups: pull.

Individual	All Actions^a^								Successful Actions^b^							
	T1	T2	T3	T4	T5	T6	B1^c^	B2^d^	T1	T2	T3	T4	T5	T6	B1^c^	B2^d^
AIS	1.00	0.75	0.60	n/a	n/a	n/a	**0.78**	**n/a**	1.00	0.50	0.60	n/a	n/a	n/a	**0.70**	**n/a**
ALB	n/a	n/a	1.00	1.00	1.00	1.00	**1.00**	**1.00**	n/a	n/a	1.00	1.00	1.00	1.00	**1.00**	**1.00**
ALE	1.00	0.80	1.00	0.80	0.75	0.67	**0.93**	**0.74**	1.00	1.00	1.00	0.75	0.80	1.00	**1.00**	**0.90**
ALM	1.00	1.00	1.00	0.97	1.00	1.00	**1.00**	**0.99**	1.00	1.00	1.00	1.00	1.00	1.00	**1.00**	**1.00**
ANG	0.50	0.00	1.00	n/a	0.43	1.00	**0.50**	**0.71**	0.50	0.00	1.00	n/a	0.60	1.00	**0.50**	**0.62**
Avg.^e^							0.84	0.86							0.84	0.88
TAN	0.71	n/a	0.50	1.00	0.40	0.38	**0.61**	**0.59**	0.67	n/a	0.67	1.00	0.00	0.40	**0.67**	**0.50**
THA	0.40	1.00	1.00	0.75	0.67	0.50	**0.80**	**0.64**	0.00	1.00	1.00	0.67	1.00	1.00	**0.67**	**0.67**
THE	0.67	0.80	1.00	0.33	0.75	0.50	**0.82**	**0.53**	0.67	n/a	1.00	0.50	0.83	0.50	**0.83**	**0.71**
TIN	1.00	1.00	1.00	1.00	1.00	1.00	**1.00**	**1.00**	1.00	1.00	1.00	1.00	1.00	1.00	**1.00**	**1.00**
TIP	1.00	n/a	1.00	n/a	1.00	0.78	**1.00**	**0.89**	1.00	n/a	1.00	n/a	1.00	0.00	**1.00**	**0.86**
TIR	1.00	1.00	1.00	1.00	1.00	n/a	**1.00**	**1.00**	1.00	1.00	1.00	1.00	1.00	n/a	**1.00**	**1.00**
TON	1.00	1.00	1.00	1.00	n/a	1.00	**1.00**	**1.00**	n/a	1.00	1.00	1.00	n/a	1.00	**1.00**	**1.00**
TRO	n/a	0.71	n/a	1.00	n/a	1.00	**0.71**	**1.00**	n/a	1.00	n/a	1.00	n/a	1.00	**1.00**	**1.00**
Avg.^e^							0.87	0.83							0.90	0.84
NIC	1.00	1.00	1.00	1.00	1.00	n/a	**1.00**	**1.00**	1.00	1.00	1.00	1.00	1.00	n/a	**1.00**	**1.00**
NIL	1.00	1.00	1.00	1.00	1.00	n/a	**1.00**	**1.00**	1.00	1.00	1.00	1.00	1.00	n/a	**1.00**	**1.00**
NOR	n/a	n/a	n/a	n/a	n/a	1.00	**n/a**	**1.00**	n/a	n/a	n/a	n/a	n/a	n/a	**n/a**	**1.00**
NAT	n/a	n/a	n/a	1.00	0.50	0.95	**n/a**	**0.82**	n/a	n/a	n/a	n/a	1.00	1.00	**n/a**	**1.00**
Avg.^e^							1.00	0.95							1.00	1.00
**Overall** f							**0.91**	**0.85**							**0.91**	**0.90**

a Proportion of pull actions in all actions.

b Proportion of pull actions in successful actions.

c Proportion of pull actions for T1–T3.

d Proportion of pull actions for T4–T6.

e Group average for blocks.

f Average across all animals in pull condition.

In order to assess whether the level of participation, measured as the number of actions at the box during all training sessions, as well as age class or group membership had an effect on the strength of preference for the trained action in the test, we calculated the correlation to each factor. None of the factors rendered significant effects on the strength of preference (Spearman rank correlations: all r<0.23, all p>0.05). Sex also did not have an effect (Mann-Whitney U-test: n_f_ = 13, n_m_ = 16, Z = 0.39, p = 0.697).

Three animals that were present but had not successfully participated in the training (NAT, TRO, WOT; marked with asterisks in Figs. 1 & 2), did succeed during test sessions and all did so using the action established in their respective group. One animal (VIK; marked with asterisks in Figs. 1 & 2) immigrated into the M-group between the first and the second test blocks and was therefore naïve to the task. In the second test block, he also succeeded using the action that had been trained by the group it joined. Therefore all four animals that did not succeed in the training adopted the trained action in the test (Binomial test: n = 4, exp. proportion = 0.5, p = 0.06, Figs. 1 & 2, Tabs. 1 & 2).

### Free Condition

In the three free condition groups, the animals could choose either the push or the pull method to successfully acquire the reward in every attempt, throughout the entire experiment. Again, participation was not evenly distributed among group members. In two groups, the dominant female produced the majority of actions and claimed most rewards. Similar to the constrained groups, juvenile or subadults were the first to manipulate the apparatus in all groups, and to succeed in two out of the three groups (Tab. S2).

**Figure 3:**
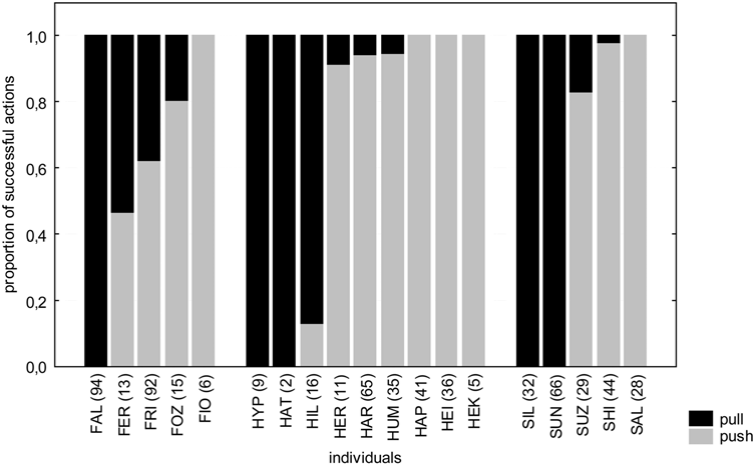
Free condition. proportion of successful actions push (grey bars) and pull (black bars) in groups. Numbers in brackets indicate number of successful manipulations.

When all performed actions were analysed, there was no general preference for push or pull across the three groups. Ten individuals showed a push preference, eight a pull preference and one individual had no preference for either method (Tab. 3, Fig. 3). Sixteen animals showed the same or an even stronger preference, when only successful actions were considered (Tab. 3, Fig. 3). Only two animals changed their preferences across time (FER, FOZ) and they did so against the predominant group preference in terms of majority of animals or the number of successful actions. Of the fifteen animals that applied both methods during the experiment, thirteen showed a significant preference for the action they were first successful with (Binomial test: exp. proportion: 0.5, p<0.01).

**Table 3:** Free condition: Number of push and pull actions for ‘all actions’ and ‘successful actions’.

Individual	All Actions				Successful Actions			
	Push^a^	Pull^a^	% Push^b^	p^d^	Push^a^	Pull^a^	% Push^b^	p^d^
**FAL (ps)**	4	90	4.26%	<0.001	0	31	0.00%	<0.001
*FER (ps)*	8	11	42.11%	0.324	6	7	46.15%	0.71
**FIO (pl)**	6	1	85.71%	0.063	6	0	100.00%	0.016
*FOZ (pl)*	20	7	74.07%	0.01	12	3	80.00%	0.018
**FRI (pl)**	70	137	33.82%	<0.001	57	35	61.96%	0.014
Avg.^c^			47.99%				57.62%	
**HAP (ps)**	67	4	94.37%	<0.001	41	0	100.00%	<0.001
**HAR (ps)**	85	10	89.47%	<0.001	61	4	93.85%	<0.001
**HAT (pl)**	0	4	0.00%	0.063	0	2	0.00%	0.25
**HEI (ps)**	45	0	100.00%	<0.001	36	0	100.00%	<0.001
**HER (ps)**	13	1	92.86%	<0.001	10	1	90.91%	0.006
**HEK (ps)**	6	0	100.00%	0.016	5	0	100.00%	0.031
**HIL (pl)**	8	30	21.05%	<0.001	2	14	12.50%	0.002
**HUM (ps)**	40	17	70.18%	0.002	33	2	94.29%	<0.001
**HYP (ps)**	3	12	20.00%	0.018	0	9	0.00%	0.002
Avg.^c^			65.33%				65.73%	
**SAL (ps)**	39	0	100.00%	<0.001	28	0	100.00%	<0.001
**SHI (ps)**	76	30	71.70%	<0.001	43	1	97.73%	<0.001
**SIL (pl)**	4	49	7.55%	<0.001	0	32	0.00%	<0.001
**SUN (pl)**	9	105	7.89%	<0.001	0	66	0.00%	<0.001
**SUZ (ps)**	35	21	62.50%	0.041	24	5	82.76%	<0.001
Avg.^c^			49.93%				56.10%	

a Absolute numbers of pushing and pulling for ‘all actions’ and ‘successful actions’.

b percentages of ‘push’ in relation to the sum of pushing and pulling behaviours.

c average percentage across individuals of a group.

d calculation of significance of preference: binomial test p<0.05; expected proportion 0.5.

Action type with which the animal was first successful with is indicated in bracket: ps – push, pl – pull.

Animal names in bold indicate preference for the action the individual was first successful with.

Groups are indicated by the first letter of an individual's acronym. Group averages are shown below each group.

## Discussion

The fact that marmosets tested in the constrained condition as well as the individuals tested in the free condition showed a significant preference for the action they were trained or first successful with, suggests that they did not conform to group norms. More specifically, the constrained groups adopted the method they had acquired during training, independent of action type, what group they were a member of or what age and sex class they belonged to. The adopted method was also maintained for one month, despite the fact that another opening method became available and was discovered in five of the six groups of the constrained condition.

In the free condition, we found no general preference for the pushing or pulling action in free-living marmosets. Unlike the control groups in the Bugnyar and Huber [29] study, the control groups displayed pulling behaviour at similar rates as pushing behaviour. Some group differences emerged in the free condition but depended more on individual preferences than on social influence. The only two animals that reversed their initial preference during the course of the experiment did so by shifting away from the group norm, to the action used by the minority. If social conformity were to apply to the preference formation in our study, one would expect the exact opposite, namely that the animals that find themselves in the minority would overcome their initial preference in order to adhere to the norm of the group. Instead, thirteen of the fifteen animals in the free condition that discovered both opening methods showed a significant preference for the action they were first successful with, independent of what the group norm was.

Clearly, our data do not support the idea that marmosets display social conformity in terms of an exaggerated preference for the behaviour of the group majority [24]. This conformist transmission would minimise differences within groups and stabilise intergroup variation. This was not observed in the groups tested in the free condition. Rather, the differences within the groups tended to increase. In humans, punishment and coercion play a crucial role in the establishment of group norms [38], but no such interaction was witnessed in our experiment and these mechanisms are highly unlikely to occur in a socially tolerant species such as marmosets [30].

What mechanisms other than conformity could lead to group differences in terms of behavioural patterns? An intriguing alternative to invoking a socially and cognitively complex phenomenon such as conformity is to focus more on the behaviour of the individuals involved. A true conformist would have to understand its role in the group, scan the group to detect the current norm and alter its personal preference. More parsimoniously, an individual could witness a first encounter with a novel feeding opportunity, acquire some social information about the food resource or how to access it and from that point on develop a routine or habit that is reinforced with every reward obtained.

Habits are routines of behaviour or learned dispositions to repeat past performances triggered by similar contexts, locations and social environments. This disposition is promoted by associative learning, strengthened when repeatedly rewarded and in some form shared across mammalian species [39]. The majority of the repertoire displayed in the daily life of humans consists of habitual behaviour, this allows multitasking or performance of actions in an automatic fashion [40], [41]. Habit formation allows past consequences to select and shape future responses to the same context [39]. This reinforcement process can lead to stable behavioural patterns, for example in the feeding position of great tit parents at the nest [42]. Dickinson [43] showed that habit formation in rats (*Rattus norvegicus*) is more likely to occur after extended training and reinforcement. The more the rats were trained to respond to a certain context, the more independent their performance became of the current value of the goal or reinforcer – a hallmark of habitual behaviour. Similarly, a recent study with young chimpanzees showed that through habit formation the acquisition of similar, but potentially more effective behaviour was inhibited [25].

Habit formation also leads to facilitating effects of contexts on the speed or accuracy of responding to stimuli [44]. The initial goal of a repeated action loses its importance and the response is triggered by the context alone. This shift is even seen in the pattern of neurotransmitter activity, when monkeys learn a context-response association. Dopamine release that was initially elicited by the reward for a specific action (lever-pushing) in a certain context (light) was later elicited by the perception of the context itself, paired with an automated response by the subject [45]. The marmosets in our study increased their efficiency throughout the course of the experiment, reducing the number of unnecessary actions and increasing their reward rate, creating a positive feedback loop for the preferred behaviour.

Undoubtedly, social influences play a role in the spread of innovations and common marmosets have been studied extensively concerning their social learning abilities [28]–[34]. Despite the fact that our study did not explicitly focus on the transmission of behaviours between individuals or groups, we found evidence for the contribution of social learning in solving the task in some individuals. The four animals that succeeded only in the test trials of the constrained groups adopted the method that had been established as the group norm, even though they had never succeeded in opening the box during the training. One individual that migrated into one of our constrained groups after test sessions had already started only performed the action used by other group members.

An initial horizontal spread of an innovation or a temporarily constrained learning opportunity (i.e. only push leads to a reward for a week) combined with subsequent habit formation could go a long way in explaining stable behavioural differences on the group level. It could give a structured group a uniform appearance solely based on the positive feedback loop created by the reward with no need for social factors such as conformity. If an action sequence consistently leads to a reward, maintaining flexibility towards social influences seems non-adaptive. Habits reduce the amount of conscious decisions, consistent monitoring and flexibility towards behaviour-changing interventions. It would not make sense to consider the social context anew every time a stimulus is presented. Overall, the added experiential aspect arising from the social context can channel and scaffold individual efforts to acquire expertise; the social learning process is therefore one of behavioural generation, not transmission [5]. Such a scenario could also account for the data in the study by Whiten and colleagues [17]. The chimpanzees would initially acquire a skill by watching a conspecific and then form a habit using the method that they were initially more successful with, without referencing their behaviour to perceived group norms.

Despite a plethora of experiments that focus on the transmission of information or behaviour patterns throughout and in between groups, there is still a gross lack of naturalistic studies with free-living animals that are tested in groups [18]. This study therefore marks the first step in an important direction and should lead to subsequent studies with a similar focus.

Our study shows that individual preferences that may stem from habit formation can largely determine the consistent behaviour of an individual across time. Only a small window exists for social input during the initial spread of a skill. Thus, while social conformity has been shown to play a major role in maintaining traditions in humans, there is not sufficient animal data to safely expand this finding beyond. Future studies may extend our approach now that the feasibility of such a field experiment has been revealed.

## Materials and Methods

This field experiment was specifically designed to test whether individual preferences are sufficient to maintain an introduced behavioural pattern and that social conformity does not represent the only mechanism that could be involved.

### Study Site & Subjects

The study was carried out in a 32 ha area of mixed primary and secondary Atlantic Forest in a private housing estate 40 km west of Recife in the state of Pernambuco, north-eastern Brazil. The initial stage of the study consisted of locating the different family groups and their respective home ranges. Fourteen family groups were located in the area and nine of those participated in the study (group size (range): 4–9, n = 57 individuals, see results and supplemental materials for details). The animals in the area are habituated to humans and can be approached to as close as one meter. Once a home range was recognized, a platform was positioned and the animals were habituated to the experimental set-up by food provisioning (apples, bananas and crackers). All animals that approached the platform were photographed and filmed repeatedly. Identification of individuals was possible due to specific face and body features, such as differences in colour and shape, scars and injuries. If two animals in a group could not be reliably distinguished, one was marked by cutting some of the hair on the tail, which had no visible effect on the animal's behaviour [35]. This study complies with Brazilian law.

### Apparatus & Set-up

**Figure 4:**
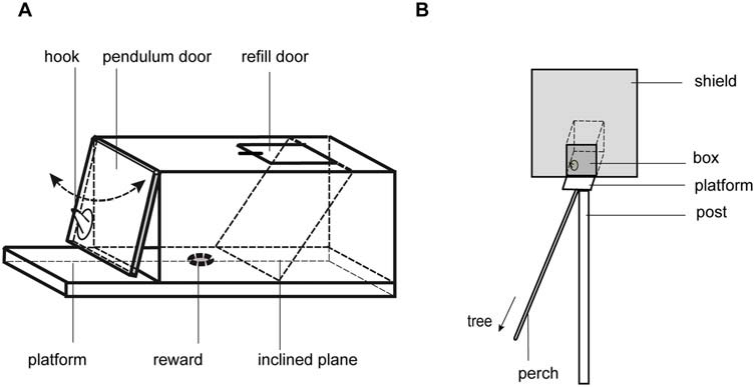
Apparatus. A: push-or-pull box (length×width×height; 20 cm×10 cm×10 cm), B: testing platform and frame (height of post: ∼130 cm).

A replica of the push-or-pull box (20×10×10 cm) designed by Bugnyar & Huber [29] was used in this study (Fig. 4a). It consisted of a wooden box that contained rewards, which could be retrieved by pulling or pushing a flap door that was attached to the front of the box. A small lockable lid on the top of the box allowed the experimenters to refill the box. The box was mounted onto the platform to which the animals had been habituated in the provisioning phase. A frame (70×70 cm) was placed near the front end of the box, to reduce the number of animals that could manipulate the box at the same time. Therefore, the marmosets could only manipulate the flap door while sitting on the platform (10×10 cm) in front of it. The platform could be reached by a perch that was connected to the closest tree (Fig. 4b).

### Experimental Conditions

We randomly assigned the nine participating groups to three different experimental conditions. Two conditions aimed at establishing one of the two opening methods within the groups (called “constrained groups”). During a training phase of twelve sessions of three trials, three of the constrained groups could only access the reward by pushing and three of the groups by pulling the flap door of the apparatus (Fig. S1, S2). A small stopper on the box restrained the animals from applying the other method. After the training phase, the animals in the constrained groups were tested by presenting the box without the stopper, so that both pushing and pulling led to reward. The first test block, consisting of three sessions, spaced at three-day intervals, was conducted immediately after the training phase. Twenty-one days after the last test session of block one, the first test session of the second block was conducted (Tab. 4).

**Table 4:** Conditions and procedure of the experiment.

Condition	CONSTRAINED						CONTROL		
Action Trained	PULL			PUSH			FREE		
Group	A	N	T	L	M	W	H	S	F
Number of Animals	7	5	8	5	4	7	9	6	6
**Training**	12 sessions (each consisting of 3 trials)						12 sessions (each consisting of 3 trials)		
**Test Block 1 (Tests 1–3)**	3 sessions (each consisting of 3 trials)								
	3 days break between each test								
**Break**	21 days								
**Test Block 2 (Tests 4–6)**	3 sessions (each consisting of 3 trials)								
	3 days break between each test								

The third condition (hereafter “free condition”) served as a control for an overall preference for one of the two actions in the study population, by allowing the animals to freely choose the opening method. The three groups in this condition received twelve sessions with the unblocked box, these were similar to the training sessions in the constrained groups (Tab. 4).

### Procedure

Once all animals had been reliably identified and habituated to the platform, each group received a single box habituation trial, in which the animals were allowed to feed out of the open box without the door being attached. After the habituation trial, the twelve training sessions consisting of three trials each were conducted, with no more than two training sessions a day, spaced at least two hours apart. A trial lasted from the first contact with the box until removal of the last food item from the box. Apple (*Malus sp*.) and banana (*Musa sp.*) pieces (approximately 10×10 mm) were used as rewards. In each trial, the number of rewards, equivalent to the number of animals in the group, was positioned in the box and then the box was made accessible to the group by removing a cloth which covered the apparatus. Sessions were only started if at least 75% of all group members were present and all animals were clearly recognized. All training and test sessions were filmed with a JVC hard drive video camera and the names and actions of the visible individuals were verbally recorded by the experimenters (T.G. and M.P.).

### Data Coding & Analysis

All training trials were coded by T.G. and M.P. in a frame by frame analysis (using Cyberlink Power Director 5.0), all test trials were coded by T.G.. The following parameters were recorded from each trial: (1) the duration of each trial, (2) the identity of the subject manipulating the box, (3) the type (“push” or “pull”) and number of actions performed by the subject (defined as door movements from the neutral vertical position), (4) the duration of the manipulation (starting with first contact with the door, ending (i) at success or (ii) when three seconds passed after unsuccessful actions ceased), (5) the number of successful openings and (6) the number of gained rewards as well as (7) the identity of monkeys nearby (within a radius of 20 cm). Reliability of coding the opening technique was assessed by parallel coding of one training trial for all nine groups by the two observers. The inter-observer agreement yielded a Cohen's Kappa of 0.975 for ‘push’ and 0.988 for ‘pull’.

Due to variation in sample size and deviation from normal distribution, non-parametric analyses were used. For all analyses p≤0.05 (two-tailed).

## Supporting Information

Figure S1 Photograph of a marmoset performing the push action during a training session
(5.57 MB TIF)

Figure S2 Photograph of a marmoset performing the pull action during a training session
(5.95 MB TIF)

Table S1 Training constrained groups: a) pull condition, b) push condition
(0.07 MB DOC)

Table S2 Free condition: participation, first contact and successful manipulation
(0.05 MB DOC)
