# Supplementary figures and images for: The Maintenance of Traditions in Marmosets: Individual Habit, Not Social Conformity? A Field Experiment
Source: PLoS One. 2009 Feb 18;4(2):e4472. doi: 10.1371/journal.pone.0004472 (PMC2636861; doi:10.1371/journal.pone.0004472)

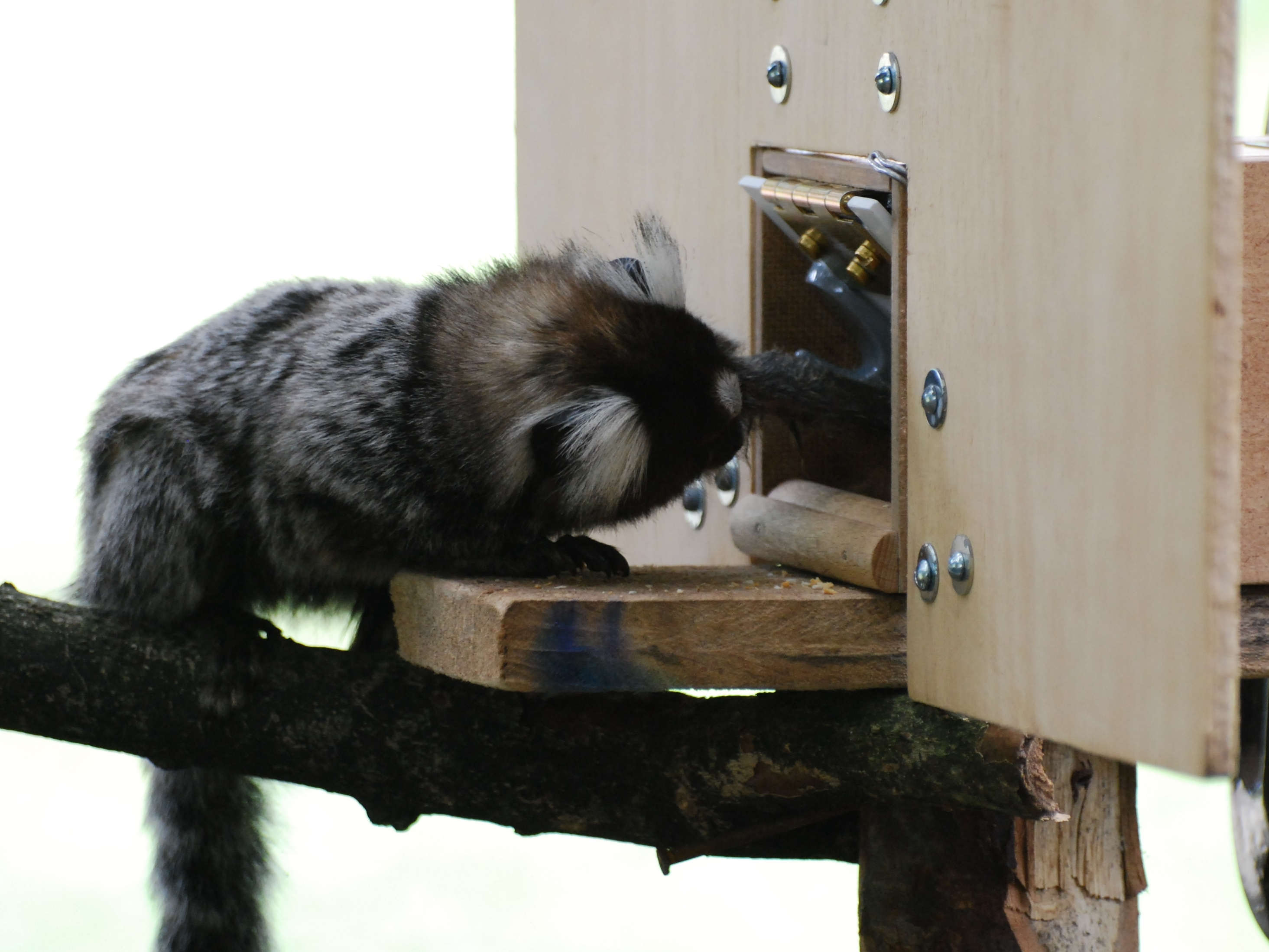

Supplement: Figure S1 — Photograph of a marmoset performing the push action during a training session (5.57 MB TIF) [file pone.0004472.s001.tif]

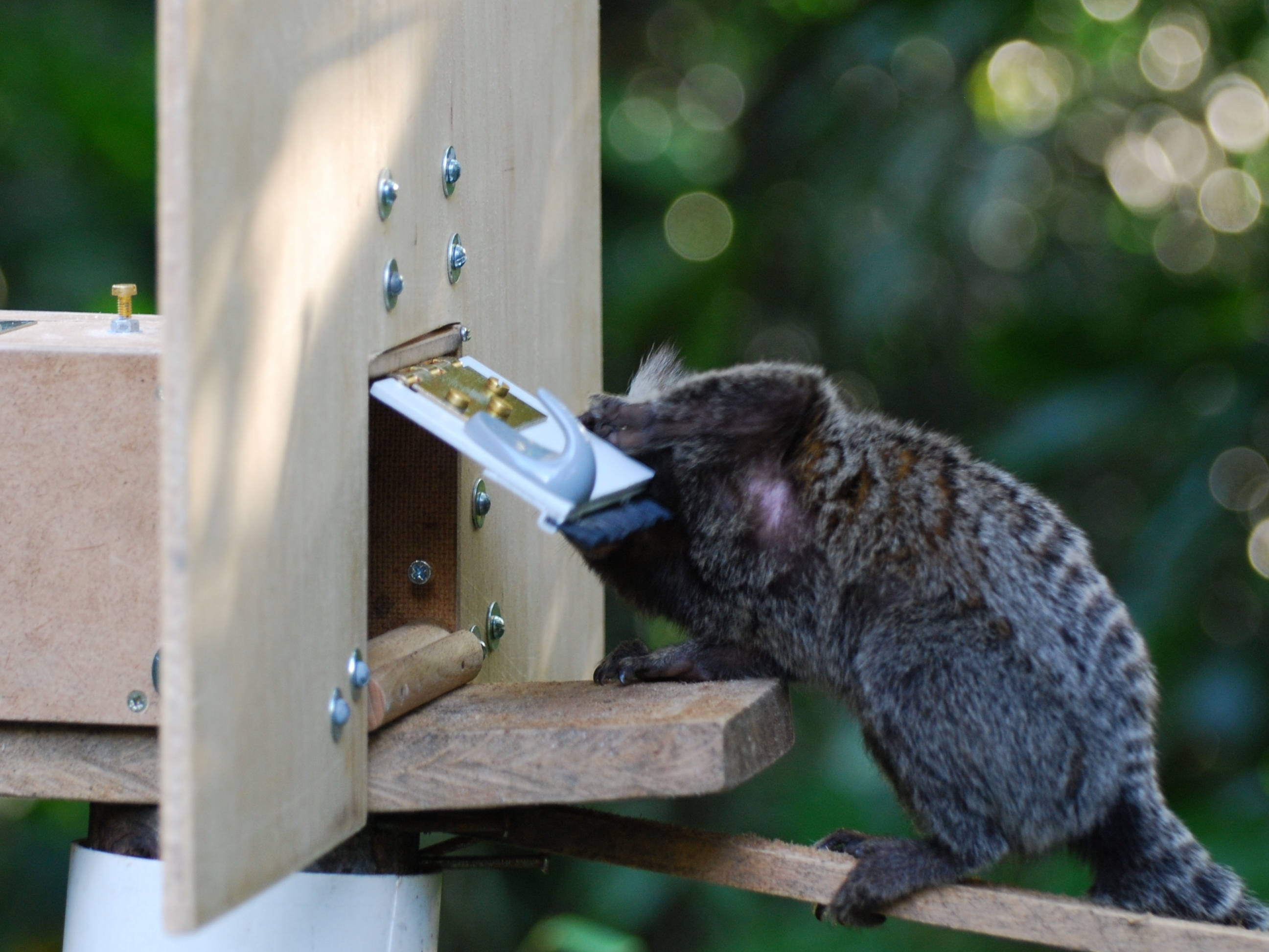

Supplement: Figure S2 — Photograph of a marmoset performing the pull action during a training session (5.95 MB TIF) [file pone.0004472.s002.tif]
