# Supplementary material for: The Maintenance of Traditions in Marmosets: Individual Habit, Not Social Conformity? A Field Experiment
Source: PLoS One. 2009 Feb 18;4(2):e4472. doi: 10.1371/journal.pone.0004472 (PMC2636861; doi:10.1371/journal.pone.0004472)
Supplement: Table S1 — Training constrained groups: a) pull condition, b) push condition (0.07 MB DOC) [file pone.0004472.s003.doc]

# The Maintenance of Traditions in Marmosets: Individual Habit, not Social Conformity? A Field Experiment.

Mario B. Pesendorfer, Tina Gunhold, Nicola Schiel, Antonio Souto, Ludwig Huber, Friederike Range

Tab. S1: Training constrained groups: a) pull condition, b) push condition

| a) | Individual | Actions ^§^ | Percent ^$^ | Sex | Age | b) | Individual | Actions ^§^ | Percent ^$^ | Sex | Age |
| --- | --- | --- | --- | --- | --- | --- | --- | --- | --- | --- | --- |
|  | ALM | 254 | 40.71% | f | a |  | LAR | 136 | 30.98% | f | a |
|  | AIS | 136 | 21.79% | f | j |  | LOR | 128 | 29.16% | f | j |
|  | ALE^#^ | 114 | 18.27% | m | j |  | LUD | 83 | 18.91% | m | a |
|  | ALB | 70 | 11.22% | m | a |  | LUN | 73 | 16.63% | f | j |
|  | ANG* | 46 | 7.37% | f | a |  | LRZ*^#^ | 19 | 4.33% | m | s |
|  |  |  |  |  |  |  |  |  |  |  |  |
|  | NIC | 142 | 46.25% | f | a |  | MAT*^#^ | 113 | 40.36% | m | s |
|  | NIL*^#^ | 75 | 24.43% | m | a |  | MAR | 94 | 33.57% | f | a |
|  | NAT | 39 | 12.70% | m | s |  | MON | 71 | 25.36% | f | s |
|  | NOR | 2 | 0.65% | m | j |  | MAO | 2 | 0.71% | m | a |
|  |  |  |  |  |  |  |  |  |  |  |  |
|  | TON^#^ | 128 | 18.26% | m | s |  | WIL*^#^ | 396 | 60.83% | m | j |
|  | THE* | 127 | 18.12% | m | j |  | WAL | 168 | 25.81% | m | j |
|  | THA | 124 | 17.69% | f | s |  | WRI | 56 | 8.60% | f | s |
|  | TIP | 86 | 12.27% | m | a |  | WOT | 22 | 3.38% | m | a |
|  | TAN | 66 | 9.42% | m | a |  |  |  |  |  |  |
|  | TIR | 66 | 9.42% | m | a |  |  |  |  |  |  |
|  | TRO | 66 | 9.42% | m | j |  |  |  |  |  |  |
|  | TIN | 38 | 5.42% | f | a |  |  |  |  |  |  |

^§^ absolute numbers of actions; ^$^ percentages of an individual's actions in relation to the sum of all actions in a group; * first contact with the box in group; ^#^ first successful manipulation in group. Groups are indicated by the first letter of an individual's acronym and separated by empty bars. (age classes: j- juvenile, s- subadult, a- adult).

# 
