# Supplementary material for: The Maintenance of Traditions in Marmosets: Individual Habit, Not Social Conformity? A Field Experiment
Source: PLoS One. 2009 Feb 18;4(2):e4472. doi: 10.1371/journal.pone.0004472 (PMC2636861; doi:10.1371/journal.pone.0004472)
Supplement: Table S2 — Free condition: participation, first contact and successful manipulation (0.05 MB DOC) [file pone.0004472.s004.doc]

# The Maintenance of Traditions in Marmosets: Individual Habit, not Social Conformity? A Field Experiment.

Mario B. Pesendorfer, Tina Gunhold, Nicola Schiel, Antonio Souto, Ludwig Huber, Friederike Range

Tab. S2: Free condition: participation, first contact and successful manipulation for each individual

| **Individual** | **Actions ^§^** | **Percent ^$^** | **Sex** | **Age** |
| --- | --- | --- | --- | --- |
| FRI | 225 | 34.56% | f | a |
| FAL*^#^ | 105 | 16.13% | f | s |
| FER | 37 | 5.68% | m | a |
| FOZ | 31 | 4.76% | m | a |
| FIO | 8 | 1.23% | f | a |
| FUN | 4 | 0.61% | m | a |
|  |  |  |  |  |
| HAR | 114 | 23.03% | f | a |
| HAP* | 107 | 21.62% | m | j |
| HUM^#^ | 76 | 15.35% | m | a |
| HIL | 63 | 12.73% | f | s |
| HEI | 61 | 12.32% | m | a |
| HER | 23 | 4.65% | f | a |
| HYP | 19 | 3.84% | m | a |
| HEK | 18 | 3.64% | m | a |
| HAT | 14 | 2.83% | m | a |
|  |  |  |  |  |
| SUN | 66 | 33.17% | m | s |
| SHI*^#^ | 44 | 22.11% | f | j |
| SIL | 32 | 16.08% | f | a |
| SUZ | 29 | 14.57% | f | a |
| SAL | 28 | 14.07% | m | j |

^§^ absolute numbers actions; ^$^ percentage of actions in relation to the sum of all actions in a group; * first contact with the box in group; ^#^ first successful manipulation in group. Groups are indicated by the first letter of an individual's acronym and separated by empty bars. (age classes: j- juvenile, s- subadult, a- adult).
